# The effects of visual design of progress indicators on time perception and emotion

**DOI:** 10.3389/fpsyg.2026.1977311

**Published:** 2026-10-23

**Authors:** Yunlong Luo, Yuhui Guo, Dengyu Wang, Yaqin Zhao, Xiaolong Lou

**Affiliations:** 1 School of Media and Design, Hangzhou Dianzi University, Hangzhou, Zhejiang, China; 2 School of Humanities and Sciences, Queen Mary University of London, London, United Kingdom

**Keywords:** conceptual metaphor theory, progress indicators, time perception, user experience, visual design

## Abstract

**Introduction:**

Computer users frequently have to wait. Well-designed progress indicators can improve the experience of passive waiting. This study examined how visual design features of progress indicators affect users’ time perception and emotional responses.

**Methods:**

Five studies examined indicator shape (Study 1), contour (Study 2), color form (Study 3), motion direction (Study 4), and concretization (Study 5). Time perception was assessed using duration estimates and 7-point ratings of perceived speed, while emotional responses were measured using an adapted Self-Assessment Manikin (SAM).

**Results:**

No significant differences were found between the bar and circle indicators. Circular contours yielded shorter duration estimates, faster perceived speed, and more positive emotional ratings than square contours. Progressive transparency change increased perceived speed compared with static color. Motion direction affected duration estimates and perceived-speed ratings, with top-to-bottom motion differing from right-to-left and bottom-to-top motion. The emotional main effect was significant, but the pairwise emotional contrast did not remain significant after Bonferroni correction. Higher concretization increased perceived-speed ratings and improved emotional evaluations, but did not affect duration estimates.

**Discussion:**

These findings show that the effects of progress-indicator design vary across outcome measures: visual features that shorten perceived duration do not necessarily improve emotional responses. We discuss the implications for interface design.

## 1 Introduction

In the process of human–computer interaction (HCI), user waiting is an inevitable and common situation. Prolonged waiting may cause some users to close the webpage (Krishnan and Sitaraman, 2012) and may also lead users to abandon transactions (Lejeune and Wearden, 2009). Informing users of loading progress during waiting is important, as it can reduce frustration and negative evaluations (Meyer et al., 1995; Bouch et al., 2000). A progress indicator is an effective design for providing informational feedback; it can alleviate anxiety and improve user experience (Lallemand and Gronier, 2012). Typically, system engineers design progress indicators in different visual forms to reduce users’ perceived waiting time. However, it remains unclear whether different visual forms in waiting interfaces affect users. Therefore, it is necessary to conduct in-depth research on progress indicators. First, based on the three types of metaphor in visual elements, we reviewed specific design methods for progress indicators. We then used five studies to measure the mechanisms through which visual elements exert their effects. Finally, based on the findings, we provide theoretical support and recommendations for interactive system developers. Our research aims to explore how visual design methods can improve users’ perception and emotion.

## 2 Related work

### 2.1 Progress indicator and time perception

Progress indicators are an important form of feedback design. They provide information about system operation and reduce users’ confusion. Early studies found that, compared with static text prompts, users prefer percentage indicators, because real-time progress information reduces uncertainty and helps users better manage their time (Myers, 1985). Visual design refers to transforming information (such as numbers and text) into visual forms, enabling users to understand complex information more intuitively. In recent years, graphical visual designs have replaced simple text/percentage-based indicators and have been widely accepted by users. One study showed that progress bars were preferred over static displays and looping indicators (Branaghan and Sanchez, 2009).

From a functional perspective, it is crucial to determine how design can reduce users’ subjective waiting time. Due to technical limitations such as hardware constraints, objective waiting time cannot yet be eliminated. However, compared with objective time, subjective waiting time has a more significant impact on customer responses (Hornik, 1984), and reducing time perception can improve user satisfaction (Pàmies et al., 2016).

### 2.2 Visual element and conceptual metaphor theory (CMT)

Among visual elements, by combining shapes through graphic design, diverse visual types can be created. Different types of progress indicators can influence users’ waiting experience, particularly in terms of shortening time perception. As visual stimuli, icons are first processed at the perceptual level and then give rise to higher-level conceptual processing. Metaphor is a highly important form of cognitive mapping in visual processing. Conceptual metaphor theory regards metaphor as a fundamental cognitive tool that people use to understand abstract phenomena (Lakoff and Johnson, 1980). When people encounter abstract concepts, they habitually understand them in terms of familiar, concrete things. Progress indicators are an example of this type of design: they attempt to construct metaphors through visual elements and thereby affect human perception. For example, when users face a waiting situation, the display of a “progress bar” can map an abstract task process onto “travelling along a straight path” allowing users to understand the system’s current operating status more easily.

Metaphors operate through different mechanisms. CMT divides metaphors into three types: structural metaphor, orientational metaphor, and ontological metaphor. They activate metaphorical meaning by producing cross-domain mappings through different mechanisms. For example, the shape of an icon may serve as a stimulus for a structural metaphor, the direction of motion of a progress indicator constitutes a condition for orientational metaphor, and the use of graphics to simulate real-world objects or human embodied experience serves as a stimulus for ontological metaphor. It should be noted that these visual representations are only sensory stimuli, and their enhancement effects may not necessarily activate metaphors (Gibbs, 2017). In other words, not all visual design methods can affect user perception, and further verification is required.

Elements presented through visual design are considered to have the potential to activate metaphors. Previous studies have investigated whether certain visual design methods affect user experience. With regard to shape, Ohtsubo and Yoshida (2014) conducted subjective evaluations of ten progress indicators and found that the length and thickness of progress bars and progress circles had no significant effect on perceived speed. Chen and Li (2020) found no difference in time perception between progress bars and progress pies, and Li et al. (2021) showed that progress bars and progress circles do not differ in time perception, although circles provide a better emotional experience. These findings indicate that progress indicators drawn using simple geometric shapes produce similar time perception, although some findings suggest that users prefer curved shapes. In addition, previous studies have mostly used dynamic graphics as stimulus materials, while shapes with fixed contours remain underexplored.

With regard to orientational metaphor, Harrison et al. (2010) found that adding guiding patterns (scrolling effects) to geometric designs could accelerate time perception. Pibernik et al. (2023) examined eight loading animations and found that the metaphorical meaning of icons had a significant impact. Their experimental materials can be regarded as constituting ontological metaphors. However, because they used indicator icons in various forms, such as dots, rings, and coffee cups, it remains unclear whether the observed effects resulted from the activation of ontological metaphor or from other factors such as graphical complexity. In their study of emotional design, Chen and Li (2020) found that cartoon icons showing task progress can accelerate time perception. However, owing to the complexity of the visual stimuli used in the experiment, it cannot be determined whether the shortening of time perception was caused by the metaphorical nature of the graphics or by other factors such as emotional identification.

Color is an important aspect of visual design. Computer systems convey visual information through output devices, making the use of color inevitable. It is well known that color has psychologically stimulating effects on individuals and can produce aesthetic responses (Schloss et al., 2015), as well as attract attention (Thönes et al., 2018). In psychology, numerous studies have examined the effects of color on time perception (Smets, 1969; Shibasaki and Masataka, 2014; Shi and Huang, 2017). In addition, color flickering has been shown to interfere with subjective time (Herbst et al., 2013; Yuasa and Yotsumoto, 2015). These findings suggest that color elements can distort time perception. In the field of progress indicators, the focus has been on the number of colors. One study found that multicolored presentations provide users with a better waiting experience than simple grayscale displays (Cheng et al., 2024). A greater number of colors provides richer information and can divert users’ attention to a greater extent. However, to our knowledge, no research has been conducted on color form that may constitute structural metaphor, and it remains unclear whether such design can influence user perception.

In summary, explorations of the visual design of progress indicators have generally focused on specific methods, such as shape and icon type. This body of research is relatively fragmented, and many methods have not been empirically tested. Therefore, this study will systematically examine and comprehensively measure visual design approaches with metaphorical potential.

### 2.3 Evaluation indicators

Time perception is a key focus in the study of waiting experience and refers to individuals’ subjective estimation of the duration of an event or the interval between two events (Huang et al., 2003). The perception of short durations is an important cognitive function that influences many aspects of human daily behavior (Zakay and Block, 1997). In human–computer interaction environments, users’ tolerance for waiting decreases significantly, and even short waiting periods can be difficult to accept (Chebat et al., 2010). Therefore, interactive system design should aim to make waiting time feel shorter and even enjoyable (Lee et al., 2017). In addition, emotion is a key factor in the interaction between individuals and their environment (Bongard et al., 2008) and can influence individuals’ estimation of time (Hornik, 1993). Visual design not only provides information about system status but can also divert users’ attention away from time or alleviate negative emotions through graphical animations. Pruyn and Smidts (1998) suggest that there is a relationship between emotion and experience, and that design elements can evoke positive emotions. The allocation of attention plays a fundamental role in cognitive load (Fang et al., 2022). The attentional gate model indicates that time perception is related to an individual’s level of attention, which is influenced by arousal (Zakay and Block, 1997). It can be inferred that stimuli with higher levels of arousal are more likely to capture attention, thereby reducing the encoding of temporal information. In addition, a large body of research shows that emotional factors such as pleasure (Angrilli et al., 1997; Droit-Volet et al., 2013) and boredom (McGuire et al., 2010) can affect time perception. Emotions also influence product satisfaction (Borges et al., 2015) and improve consumers’ evaluation of service quality (Baker et al., 1994). Overall, time perception and emotion are important dimensions for measuring users’ waiting experience.

## 3 Research objectives

This study examines how visual elements influence the waiting experience in human–computer interaction and addresses the following research questions:

(1) Does shape affect users’ time perception and emotional responses?
(2) Does contour affect users’ time perception and emotional responses?
(3) Does color form affect users’ time perception and emotional responses?
(4) Does motion direction affect users’ time perception and emotional responses?
(5) Does concretization affect users’ time perception and emotional responses?

The research model is shown in Figure 1.

**FIGURE 1:**
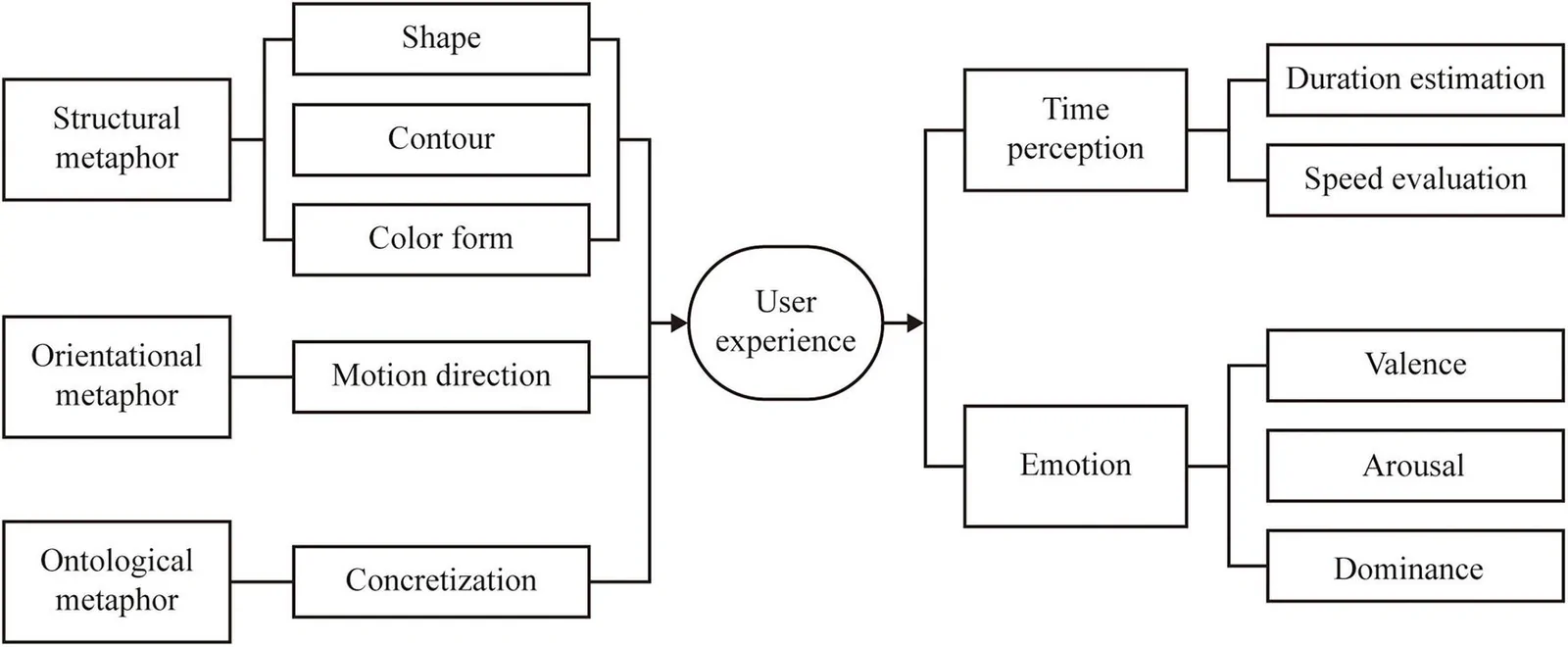
Research model.

## 4 Study 1: shape

### 4.1 Experimental design

To examine the findings concerning bar and circular designs, we measured these two shapes again to verify their effects on users’ time perception and emotion.

Participants estimated the duration of each trial. They rated perceived speed on a 7-point scale (1 = “very slow,” 7 = “very fast”). Emotional responses were measured using an adapted Self-Assessment Manikin (SAM), with 7-point ratings for valence (1 = “very unpleasant,” 7 = “very pleasant”), arousal (1 = “very bored,” 7 = “very excited”), and dominance (1 = “very helpless,” 7 = “very much in control”). The self-report items were designed following established usability principles for survey research (Geisen and Bergstrom, 2017).

### 4.2 Stimulus materials and apparatus

Two loading interfaces were created to present participants with two types of progress indicator. The video materials had a resolution of 1080p and a frame rate of 30 fps. The path length of each shape was approximately 800 pt, with an error of no more than 5 pt, and both were 15 pt wide. The bar progressed from left to right, while the circle rotated clockwise from the top. The loading time was 7 s in both conditions (Figure 2).

**FIGURE 2:**
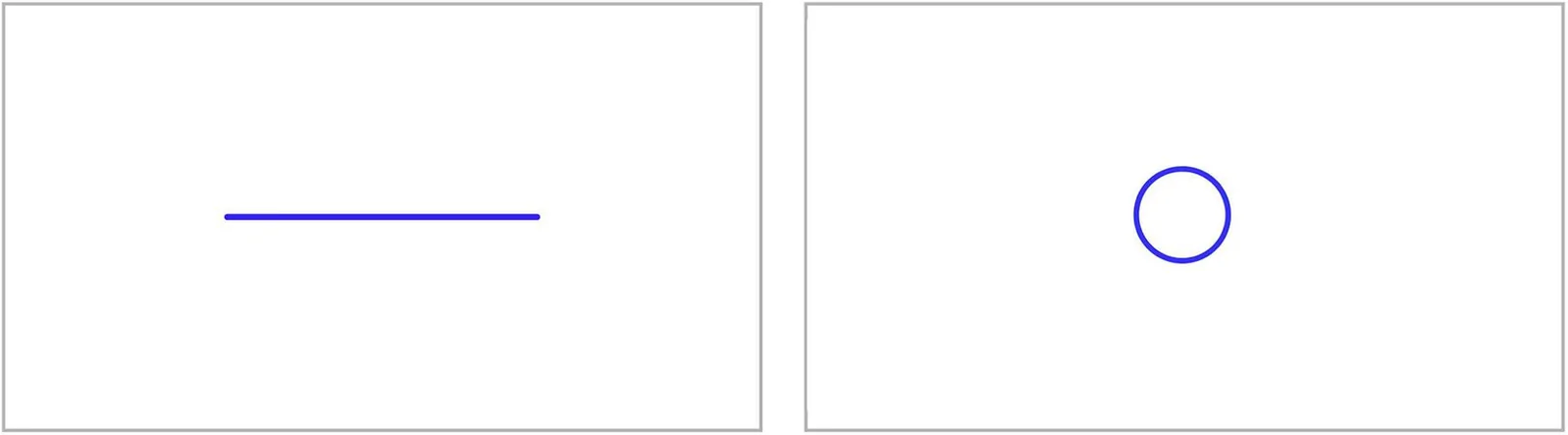
Examples of the stimulus materials used in Study 1.

The experiment was conducted on a Lenovo Y7000P IRX9 computer. The stimuli were displayed on a 15.6-inch monitor with a resolution of 1920 × 1080 pixels and a refresh rate of 60 Hz.

### 4.3 Participants

23 participants (14 females and 9 males; *M* = 23 years, SD = 1.57 years) were recruited for the experiment. All participants were highly familiar with progress indicators and were proficient in operating computer devices. All had normal or corrected-to-normal vision. The experiment was carried out in accordance with the principles of the Declaration of Helsinki. The experimental protocol was reviewed and approved by the Ethics Committee of Hangzhou Dianzi University. After the experiment, they received course credit as compensation.

### 4.4 Procedure

(1) Participants were invited to a spacious classroom. A within-subject experimental design was used. The experimenter introduced the procedure and informed participants that they would be required to provide subjective evaluations of waiting time, but should not count the seconds. Demographic information, including gender and age was then recorded.
(2) After the experimental program was launched, participants completed each step by clicking. After a stimulus had finished playing, participants answered the corresponding items and then clicked the Continue button again to display the next stimulus. This process continued until the experiment ended.

The stimuli were presented using an AB/BA design to counterbalance order effects. Participants were divided into two groups, which experienced the stimuli in opposite orders.

(3) Upon completion of the experiment, all data were organized and prepared for analysis. The entire experimental procedure lasted approximately 10 minutes.

### 4.5 Results and discussion

Data were analyzed using IBM SPSS Statistics 27. Paired-samples *t*-tests were used to analyze differences; when the data did not satisfy the assumption of normality, the Wilcoxon signed-rank test was used. The significance level was set at α = 0.05.

There was no significant difference in duration estimation between the bar (*M* = 6.35, SD = 1.75) and the circle (*M* = 7.17, SD = 2.06) [*t*(22) = −1.687, *p* = 0.106]. There was also no significant difference in speed evaluation between the bar (*M* = 4.17, SD = 0.89) and the circle (*M* = 3.78, SD = 1.31) [*t*(22) = 1.204, *p* = 0.242]. In terms of emotion, no significant difference was found between the bar (*M* = 4.09, SD = 0.85) and the circle (*M* = 4.32, SD = 0.92) [*t*(22) = −1.234, *p* = 0.230] (Tables 1, 2).

**TABLE 1:** Mean and standard deviation of the variables in Study 1.

Factor	Type	Evaluation indicator	*M*	*SD*
Shape	Bar	Duration estimation	6.35	1.75
		Speed evaluation	4.17	0.89
		Emotion	4.09	0.85
	Circle	Duration estimation	7.17	2.06
		Speed evaluation	3.78	1.31
		Emotion	4.32	0.92

**TABLE 2:** The paired sample *t*-test results from Study 1.

Factor	Evaluation indicator	*t*	*df*	*P*
Shape	Duration estimation	−1.687	22	0.106
	Speed evaluation	1.204	22	0.242
	Emotion	−1.234	22	0.230

The results for time perception are consistent with previous research (Li et al., 2021), as no significant difference was found between the two common progress-indicator shapes. No significant difference was found in emotion either. This is broadly consistent with some previous studies (Li et al., 2021), although other research has suggested that curved shapes are preferred (Chen et al., 2016). One possible explanation is that the 7-s presentation time of the experimental materials was relatively short, so participants focused on becoming familiar with the progress-loading process and overlooked the shape. Another possible explanation is that progress circles are very common in everyday life and are often accompanied by unpleasant situations such as network latency and signal loss, thereby affecting emotional evaluations.

## 5 Study 2: contour

Study 1 examined dynamically changing bar-shaped and circular progress indicators. Study 2 focused on static contour shapes, which have received less attention. Therefore, in Study 2, we selected basic geometric shapes–circular and square–as the contours for progress indicators to measure the potential effects of these two contours.

### 5.1 Experimental design

The experimental structure was identical to that of Study 1, except that the stimulus materials were loading interfaces presenting progress indicators with two different contour designs.

The measures and methods were identical to those used in Study 1.

### 5.2 Stimulus materials and apparatus

Container-type icons were used as the basic structure. The two progress indicators had identical internal fill animations and colors and differed only in whether their contours were circular or square. The icon areas were nearly identical, with a relative difference of <0.06% (Figure 3). The video materials had a resolution of 1080p and a frame rate of 30 fps. The loading time was 7 s in both conditions (Figure 3).

**FIGURE 3:**
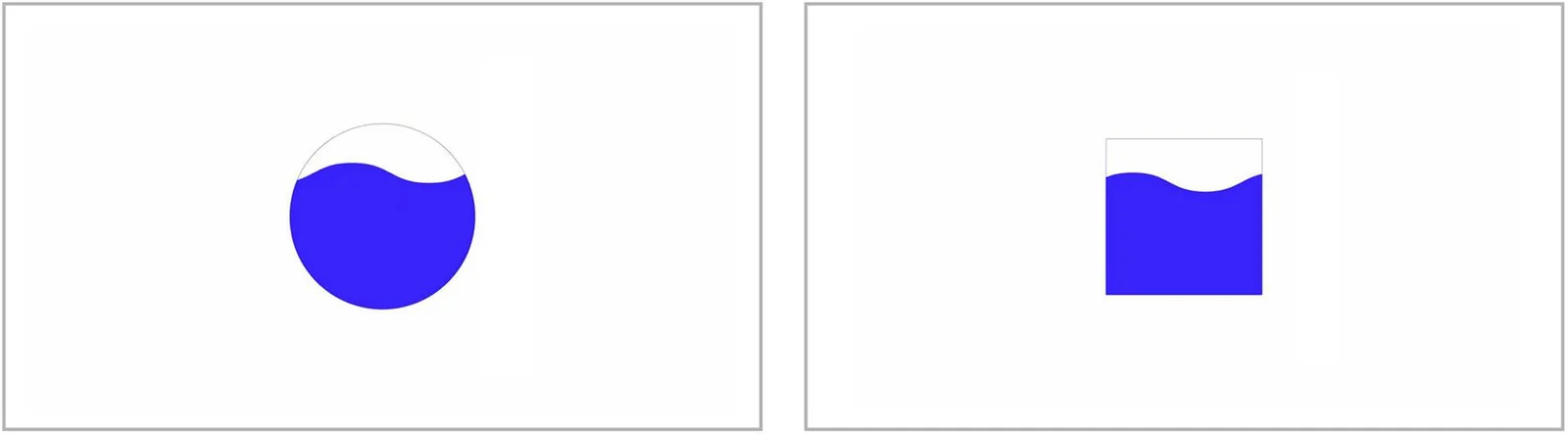
Examples of the stimulus materials used in Study 2.

The experimental equipment was identical to that used in Study 1.

### 5.3 Participants

20 participants (14 females and 6 males; *M* = 20.3 years, SD = 0.73 years) were recruited for the experiment. Other participant-related details were identical to those in Study 1.

### 5.4 Procedure

The experimental procedure was identical to that used in Study 1.

### 5.5 Results and discussion

The statistical software and methods were identical to those used in Study 1.

There was a significant difference in duration estimation between the circular contour (*M* = 5.65, SD = 1.84) and the square contour (*M* = 7.05, SD = 1.76) [*t*(19) = −4.626, *p* < 0.001], with a shorter estimated duration for the circular contour. There was also a significant difference in speed evaluation [*t*(19) = 4.761, *p* < 0.001], with the circular contour (*M* = 5.10, SD = 0.91) making time feel as though it passed faster than the square contour (*M* = 3.75, SD = 1.02). In terms of emotion, there was a significant difference between the circular contour (*M* = 5.32, SD = 1.01) and the square contour (*M* = 4.32, SD = 0.94) [*t*(19) = 6.381, *p* < 0.001], with the circular evoke more positive emotions (Tables 3, 4).

**TABLE 3:** Mean and standard deviation of the variables in Study 2.

Factor	Type	Evaluation indicator	*M*	*SD*
Contour	Circular	Duration estimation	5.65	1.84
		Speed evaluation	5.10	0.91
		Emotion	5.32	1.01
	Square	Duration estimation	7.05	1.76
		Speed evaluation	3.75	1.02
		Emotion	4.32	0.94

**TABLE 4:** The paired sample *t*-test results from Study 2.

Factor	Evaluation indicator	*t*	*df*	*P*
Contour	Duration estimation	−4.626	19	< 0.001
	Speed evaluation	4.761	19	< 0.001
	Emotion	6.381	19	< 0.001

The experimental results showed that circular contour improved time perception. One possible explanation is that, although the actual areas within the contour of the two icons were identical, a perceptual illusion may have made the circular appear larger and the square smaller. Because a larger perceived area was thought to require a faster filling speed, this may have produced a perceptual distortion (Song et al., 2025). The results also showed that the emotional evaluation of the circular contour was higher than that of the square contour. This is consistent with many previous findings. For example, Silvia and Barona (2009) found that people respond more favorably to circles than to polygons. An evolutionary-psychology perspective can explain why people prefer shapes composed of curves (Carbon, 2010). Sharp changes in contour are often associated with potential threats (Bar and Neta, 2006), whereas curved visual objects tend to evoke greater feelings of pleasure and affinity.

## 6 Study 3: color form

Color can also enhance interface aesthetics and user enjoyment. Previous research has shown that adding scrolling animation to geometric designs can create an apparent-motion effect and shorten perceived waiting time (Harrison et al., 2010). It can be argued that this color design activates directional metaphor, resulting in a distortion of time duration. However, few studies have explored how structural metaphor design of color in progress indicators affect users’perception. Therefore, in Study 3, we will measure color form that have the potential for structural metaphor.

### 6.1 Experimental design

The experimental structure was identical to that of Study 1, except that the stimulus materials were loading interfaces presenting progress indicators with two different color forms.

The measures and methods were identical to those used in Study 1.

### 6.2 Stimulus materials and apparatus

A circular container icon was used as the basic shape. The two progress indicators differed only in the way color was presented. One used static color, while the other mapped the gradual completion of the progress process by controlling progressive transparency. The color changed with progress completion, from completely transparent to completely opaque. The video materials had a resolution of 1080p and a frame rate of 30 fps. The loading time was 10 s in both conditions (Figure 4).

**FIGURE 4:**
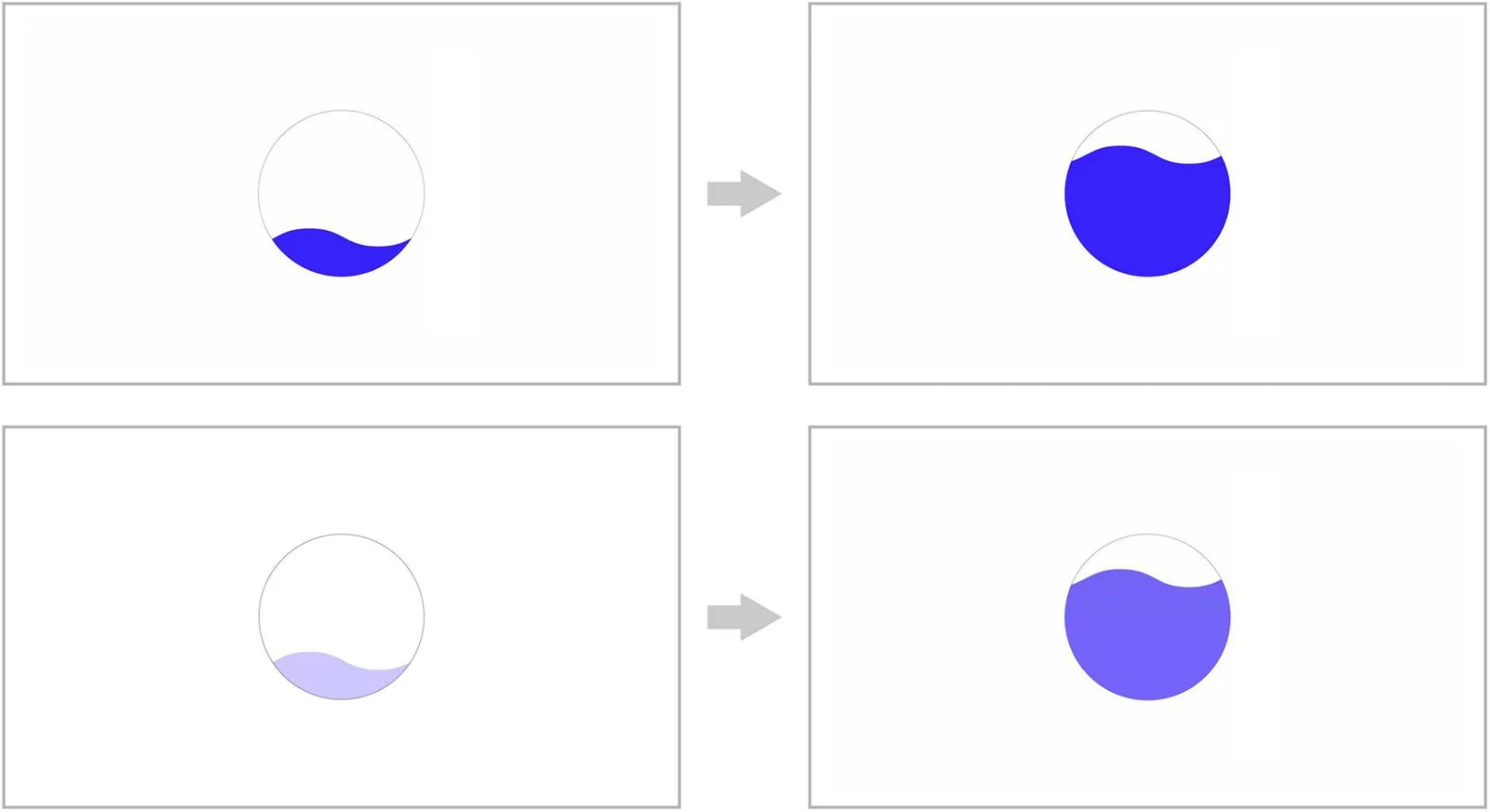
Examples of the stimulus materials used in Study 3.

The experimental equipment was identical to that used in Study 1.

### 6.3 Participants

19 participants (10 females and 9 males; *M* = 22.58 years, SD = 4.19 years) were recruited for the experiment. Other participant-related details were identical to those in Study 1.

### 6.4 Procedure

The experimental procedure was identical to that used in Study 1.

### 6.5 Results and discussion

The statistical software and methods were identical to those used in Study 1.

There was no significant difference in duration estimation between static color (Mdn = 9.43) and progressive transparency (Mdn = 9.45) [*Z* = −0.241, *p* = 0.809], whereas the difference in speed evaluation was significant [*Z* = −2.070, *p* = 0.038], with progressive transparency (Mdn = 5.00) producing a faster perception of time than static color (Mdn = 4.00). In terms of emotion, there was no significant difference between static color (*M* = 4.54, SD = 1.41) and progressive transparency (*M* = 4.88, SD = 1.39) [*t*(18) = −0.961, *p* = 0.349](Tables 5, 6).

**TABLE 5:** Mean and standard deviation of the variables in Study 3.

Factor	Type	Evaluation indicator	*M*	*SD*
Color form	Static color	Duration estimation	9.12	1.96
		Speed evaluation	4.16	0.90
		Emotion	4.54	1.41
	Progressive transparency	Duration estimation	9.45	1.93
		Speed evaluation	4.63	0.68
		Emotion	4.88	1.39

**TABLE 6:** Analysis of the differences between Static color and Progressive transparency in Study 3.

Factor	Evaluation indicator	*t*	*z*	*df*	*P*
Color form	Duration estimation	–	−0.241	–	0.809
	Speed evaluation	–	−2.070	–	0.038
	Emotion	−0.961	–	18	0.349

Experimental results indicate that color form with potential structural metaphor significantly affect perceived speed, making the waiting process feel faster. The phenomenon of time compression can be explained by the attentional gate model (Block et al., 2010). The attentional gate model proposes that the human body contains an internal pacemaker that controls pulses; when a person is aroused by external stimuli, the pacemaker speeds up and produces more pulses. These pulses then encounter a “gate,” whose opening and closing are influenced by attention to time. The less attention a person pays to temporal information, the smaller the gate opening, allowing fewer pulses to pass through and thereby shortening time perception (Grondin, 2010). Based on this model, we can infer that, through continuous updates to transparency, a progress indicator can continuously attract users’ attention and thus divert their attention away from time. Static color, by contrast, may be unable to produce a sustained diversion of attention because users quickly recognize the color setting. There was no significant difference between the two designs in their effect on emotion. This may be due to the relatively simple changes in color of the stimuli, which did not elicit differences in emotional evaluation.

## 7 Study 4: motion direction

In Study 4, we measured four types of progress indicator with different directions of motion.

### 7.1 Experimental design

The experimental structure was identical to that of Study 1, except that the stimulus materials were loading interfaces presenting progress indicators with four different directions of motion.

The measures and methods were identical to those used in Study 1.

### 7.2 Stimulus materials and apparatus

A progress bar was used as the basic icon. The four progress bars moved from left to right, right to left, bottom to top, and top to bottom, respectively. All four progress bars had the same length, width, and color. The video materials had a resolution of 1080p and a frame rate of 30 fps. The loading time was 7 s in all conditions (Figure 5).

**FIGURE 5:**
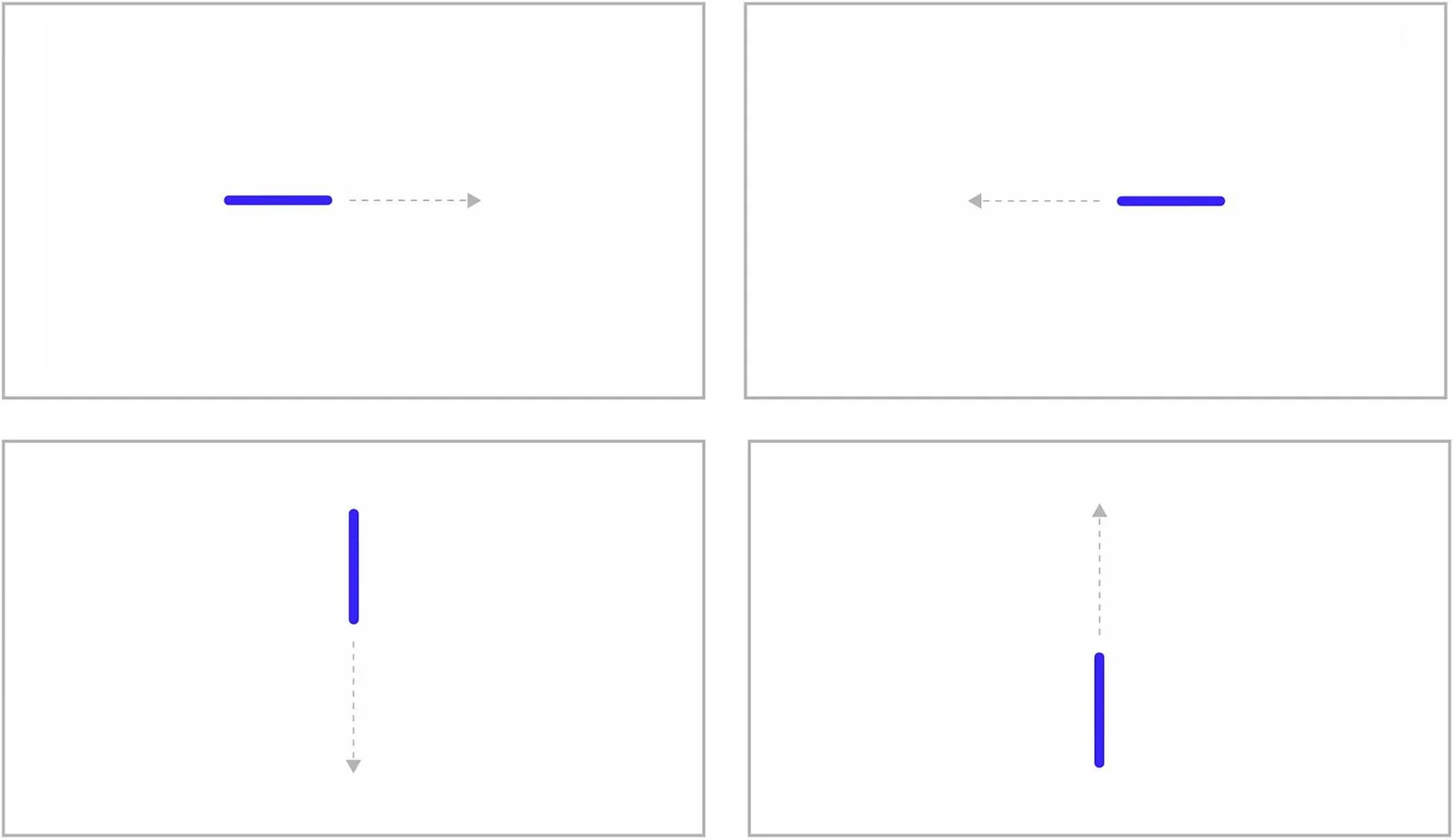
Examples of the stimulus materials used in Study 4.

The experimental equipment was identical to that used in Study 1.

### 7.3 Participants

43 participants (28 females and 15 males; *M* = 21.74 years, SD = 1.84 years) were recruited for the experiment. Other participant-related details were identical to those in Study 1.

### 7.4 Procedure

Unlike Study 1, the present experiment used a 4 × 4 Latin square design to counterbalance order effects. Participants were randomly assigned to one of the sequence groups. All other aspects were identical to those of Study 1.

### 7.5 Results and discussion

Data were analyzed using IBM SPSS Statistics 27. Repeated-measures analysis of variance was used to test the four levels. If the main effect was significant, *post hoc* comparisons were conducted with Bonferroni correction. If the data did not satisfy the assumption of normality, the Friedman test was used to examine the overall effect. If the overall effect was significant, *post hoc* pairwise comparisons were conducted using the Wilcoxon signed-rank test with Bonferroni correction. The significance level was set at α = 0.05.

Motion direction significantly affected duration estimation [χ^2^(3) = 23.104, *p* < 0.001]. Bonferroni-adjusted Wilcoxon signed-rank tests (α = 0.0083) showed differences between right-to-left and top-to-bottom motion (Mdn = 6.00 vs. 8.00, *Z* = −3.953, *p* < 0.001) and between bottom-to-top and top-to-bottom motion (Mdn = 7.00 vs. 8.00, *Z* = −3.005, *p* = 0.003). Motion direction also significantly affected speed evaluation [χ^2^ (3) = 13.869, *p* = 0.003]. *Post hoc* comparisons showed differences between right-to-left and top-to-bottom motion (Mdn = 4.00 vs. 3.00, *Z* = −3.015, *p* = 0.003) and between bottom-to-top and top-to-bottom motion (Mdn = 3.00 in both conditions, *Z* = −3.133, *p* = 0.002). For emotional ratings, a repeated-measures ANOVA showed a significant main effect of motion direction, [*F*(3, 126) = 2.729, *p* = 0.047, η^2^p = 0.061]. The pairwise comparison between bottom-to-top (*M* = 3.80, SD = 0.91) and top-to-bottom motion (*M* = 3.36, SD = 0.83) was not significant after Bonferroni correction (reported *p* = 0.035; adjusted α = 0.0083) (Tables 7, 8).

**TABLE 7:** Mean and standard deviation of the variables in Study 4.

Factor	Type	Evaluation indicator	*M*	*SD*
Motion direction	Left to right	Duration estimation	7.12	1.83
		Speed evaluation	3.35	0.97
		Emotion	3.67	0.95
	Right to left	Duration estimation	6.50	2.04
		Speed evaluation	3.58	0.93
		Emotion	3.51	0.88
	Bottom to top	Duration estimation	7.05	2.01
		Speed evaluation	3.53	0.98
		Emotion	3.80	0.91
	Top to bottom	Duration estimation	7.84	2.32
		Speed evaluation	2.95	0.98
		Emotion	3.36	0.83

**TABLE 8:** Analysis of differences among four motion directions in Study 4.

Factor	Evaluation indicator	*F*	χ^2^	*df*	*P*	*Post-hoc* comparison test
Motion direction	Duration estimation	–	23.104	3	< 0.001	Top-to-bottom > right-to-left; Top-to-bottom > bottom-to-top
	Speed evaluation	–	13.869	3	0.003	Right-to-left < top-to-bottom; Bottom-to-top < top-to-bottom
	Emotion	2.729	–	3	0.047	Bottom-to-top < top-to-bottom

Top-to-bottom motion was associated with longer duration estimates and lower perceived-speed ratings than right-to-left and bottom-to-top motion. Its emotional rating was lower than that for bottom-to-top motion, but this pairwise difference did not remain significant after Bonferroni correction. One possible explanation is symbolic speed theory (Song et al., 2025): when motion implies greater speed, users may perceive time as passing more slowly, whereas motion implying lower speed may make time seem to pass more quickly. The perceived speed of vertical descent may therefore distort time perception even when the loading duration is held constant.

## 8 Study 5: concretization

Concretization involves treating abstract concepts as entities composed of matter or other substances. In Study 5, we measured designs with two levels of concretization.

### 8.1 Experimental design

The experimental structure was identical to that of Study 1, except that the stimulus materials were loading interfaces presenting progress indicators with two levels of concretization.

The measures and methods were identical to those used in Study 1.

### 8.2 Stimulus materials and apparatus

In this experiment, we created the stimulus materials using different lines. A circular container icon was used as the basic graphic. The two progress indicators had identical contours, areas, and colors. The findings of Study 1 showed no significant differences between straight-line and curved-line structures in user perception or emotion during loading waits. Therefore, we used two types of line to distinguish the levels of metaphor. In one progress indicator, a straight line marked the fill level of the container, whereas the fill boundary of the other used a more concrete contour that simulated flowing water. The two progress indicators were considered to have low and high levels of metaphorical potential, respectively. The video materials had a resolution of 1080p and a frame rate of 30 fps. The loading time was 7 s in both conditions (Figure 6).

**FIGURE 6:**
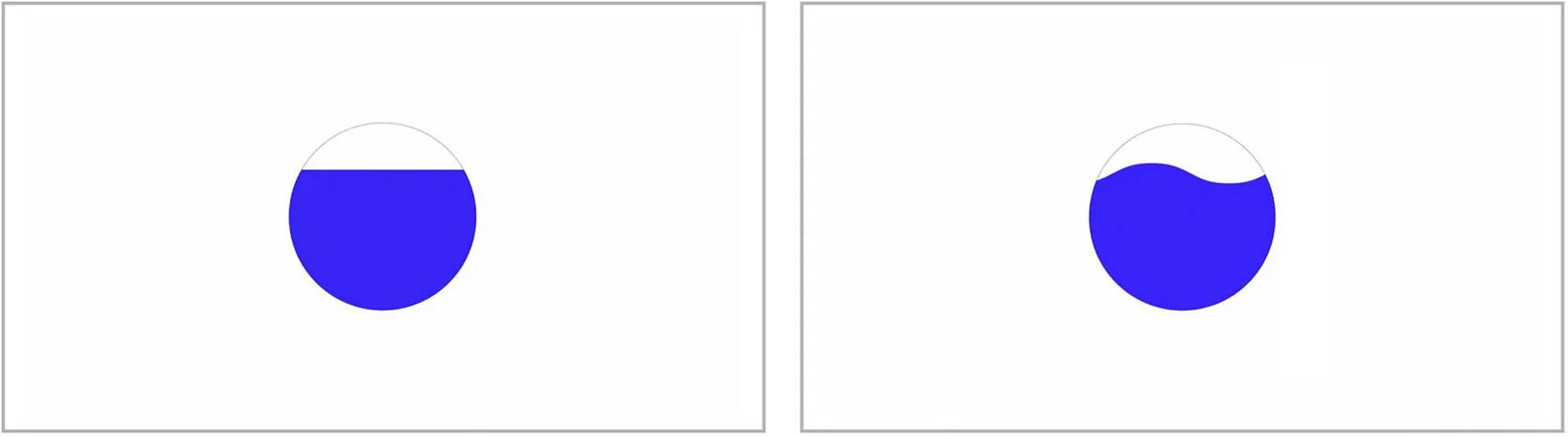
Examples of the stimulus materials used in Study 5.

The experimental equipment was identical to that used in Study 1.

### 8.3 Participants

20 participants (14 females and 6 males; *M* = 20.3 years, SD = 0.73 years) were recruited for the experiment. Other participant-related details were identical to those in Study 1.

### 8.4 Procedure

The experimental procedure was identical to that used in Study 1.

### 8.5 Results and discussion

The statistical software and methods were identical to those used in Study 1.

Concretization had no significant effect on duration estimation [*t*(19) = 0.698, *p* = 0.494], but the difference in speed evaluation was significant [*Z* = −2.038, *p* = 0.042], with the high-level condition (Mdn = 5.00) producing a faster perceived speed than the low-level condition (Mdn = 4.50). The difference in emotion was also significant [*Z* = −3.316, *p* < 0.001], with the high-level condition (Mdn = 5.00) producing a more positive emotional experience than the low-level condition (Mdn = 3.67) (Tables 9, 10).

**TABLE 9:** Mean and standard deviation of the variables in Study 5.

Factor	Level	Evaluation indicator	*M*	*SD*
Concretization	Low-level	Duration estimation	5.85	1.87
		Speed evaluation	4.40	1.19
		Emotion	4.15	1.16
	High-level	Duration estimation	5.65	1.84
		Speed evaluation	5.10	0.91
		Emotion	5.32	1.01

**TABLE 10:** Analysis of the differences between low-level and high-level in Study 5.

Factor	Evaluation indicator	*t*	*z*	*df*	*P*
Concretization	Duration estimation	0.698	–	19	0.494
	Speed evaluation	–	−2.038	–	0.042
	Emotion	–	−3.316	–	< 0.001

The experimental results showed that designs with more concrete characteristics of real-world objects can improve the waiting experience. This result confirms the key role of metaphorical elements in waiting experience (Pibernik et al., 2023). Both designs were familiar to participants, but the higher-metaphor design may have activated experiential associations more readily and thereby diverted attention. The results also suggest that a higher degree of graphical metaphor can improve emotional responses. Appealing metaphors enhance the enjoyment of interactive interfaces (Shneiderman, 2004). However, duration estimation was unaffected. This may be because the identical contour shapes allowed participants to estimate the remaining distance to be filled quickly, so the changing water-flow graphic did not distort time perception during loading. In addition, our stimulus materials were created using simple lines and flat color fills and did not involve more complex skeuomorphic icons. The conclusions of this experiment should therefore be interpreted with caution. For example, the storage size model suggests that an increase in the amount of information processed within a target duration leads to a corresponding increase in time estimation (Robert, 1969). In other words, we cannot simply assume that greater complexity results in shorter time perception. While various design approaches can be used to shift users’ attention, it is also necessary to consider whether the complexity of elements may impose excessive information processing demands on users.

## 9 Conclusion

Progress indicators are important interface elements in computer systems. It is used to prompt the system loading process and improve the user’s mood. Visual design elements alter user perception by constructing metaphors. This study experimentally measured five visual design approaches for progress indicators, identifying the mechanisms by which visual elements activate three metaphorical structures and achieving its research objectives. The aim was to determine which visual elements or design methods could enhance the perceived performance of progress indicators. Across five experiments, we first replicated prior findings on shape, then explored contour, color form, motion direction, and concretization. The results show that different visual design methods have varying effects on the perceived performance of progress indicators, with contour, color form, motion direction, and concretization. Significantly influencing time perception, while contour, motion direction, and concretization notably affect users’emotional evaluations.

## 10 Limitations and industry recommendations

This study has certain limitations. First, this study selected progress bars and progress circles as experimental materials. However, contemporary visual design includes a wide variety of styles, and future research could examine a broader range of elements. Second, psychological literature has confirmed the effect of color on time perception, and future research could investigate specific color variations in more detail. In addition, variables such as the frequency of change in dynamic colors may influence user experience, and these aspects will be explored further in future work. Finally, in this study, the experimental setting required participants to directly experience the operation of a progress-loading interface without a simulated user scenario, which differs from real-world computer-use settings. Future research could improve the research procedure by simulating everyday use scenarios.

Although our study has certain limitations, its findings allow us to offer the following recommendations for relevant industries:

(1) Circular contours were associated with shorter duration estimates and more positive emotional ratings than square contours in this study. Designers may consider circular contours when they fit the interface context;
(2) Progressive transparency increased perceived-speed ratings relative to static color in this study. Designers may use progressive transparency changes to communicate loading progress and help waiting feel shorter;
(3) In different motion directions, from top to bottom should be avoided as much as possible;
(4) Progress indicators with higher levels of concretization elicited faster perceived speed and more positive emotional evaluations, although duration estimates were unchanged. Designers may consider concrete visual cues while keeping the display simple enough to avoid excessive information processing.

## Data Availability

The raw data supporting the conclusions of this article will be made available by the authors, without undue reservation.
